# Mapping the Probability of Schistosomiasis and Associated Uncertainty, West Africa

**DOI:** 10.3201/eid1410.080366

**Published:** 2008-10

**Authors:** Archie C.A. Clements, Amadou Garba, Moussa Sacko, Seydou Touré, Robert Dembelé, Aly Landouré, Elisa Bosque-Oliva, Albis F. Gabrielli, Alan Fenwick

**Affiliations:** University of Queensland, Herston, Queensland, Australia (A.C.A. Clements); Ministère de la Santé Publique et de la Lutte Contre les Endémies, Niamey, Niger (A. Garba); Ministère de la Santé, Bamako, Mali (M. Sacko, R. Dembelé, A. Landouré); Ministère de la Santé, Ouagadougou, Burkina Faso (S. Touré); Imperial College, London, UK (E. Bosque-Oliva, A. Fenwick); World Health Organization, Geneva, Switzerland (A.F. Gabrielli)

**Keywords:** uncertainty, spatial analysis, schistosomiasis, Bayesian methods, dispatch

## Abstract

We aimed to map the probability of *Schistosoma haematobium* infection being >50%, a threshold for annual mass praziquantel distribution. Parasitologic surveys were conducted in Burkina Faso, Mali, and Niger, 2004–2006, and predictions were made by using Bayesian geostatistical models. Clusters with >50% probability of having >50% prevalence were delineated in each country.

Large-scale control programs for tropical infectious diseases have been initiated in recent years (*1*,*2*), after renewed commitment by governments and international funding agencies to support the control of previously neglected tropical diseases, including parasitic diseases such as malaria, schistosomiasis, onchocerciasis, lymphatic filariasis, and soil-transmitted helminth infections. Schistosomiasis is the second-most important parasitic disease throughout the world, with an estimated 207 million persons infected (*3*).

Success and sustainability of large-scale disease control programs depend on the allocation of resources where they will have maximum benefit (*4*). Given that tropical infectious diseases, such as schistosomiasis, tend to occur in spatially defined foci (i.e., clusters or hot spots) (*5*), efficient resource allocation relies on identifying the location of high-risk populations. Because disease-endemic countries do not have sophisticated surveillance systems that can accurately delineate disease clusters, alternative methods such as sample-based spatial prediction need to be applied to target control programs.

Among the multinational and multi-institutional partnerships formed to confront the problem of neglected tropical diseases is the Schistosomiasis Control Initiative (SCI; www.schisto.org), which supports national schistosomiasis and soil-transmitted helminth control programs in Burkina Faso, Mali, and Niger (and other African countries). Lengeler et al. (*6*) describe 2 approaches to targeting interventions: one in which the number of recipient schools or communities is determined by available resources and the other in which a prevalence threshold is defined above which all schools or communities benefit from the intervention. SCI takes the latter approach, delineating areas according to the World Health Organization (WHO)–recommended threshold prevalence of 50% for annual mass treatment. However, even this approach needs to take into account factors such as resource availability and decision risk because uncertainties exist when delineating areas based on the selected threshold.

Knowledge of uncertainty regarding the location and spatial dimensions of clusters is important because it makes possible a prior assessment of the risks and potential consequences associated with different resource allocation strategies. Uncertainties in spatial prediction maps originate from factors such as natural random variation and measurement error of the outcome variable and covariates. Bayesian methods are useful because they provide an approach for propagating uncertainty (through a prediction model) in regards to the spatial predictions. Only recently have practical applications of Bayesian methods in large-scale tropical disease control programs been reported (*7*–*9*).

## The Study

The objective of this study was to produce maps that could be integrated into the SCI-supported national intervention strategies and that explicitly represent uncertainties in spatial predictions so that national control managers could judge the quality of the evidence upon which the strategies will be based. The SCI-supported programs involve mass distribution of praziquantel (for urinary and intestinal schistosomiasis) and albendazole (for soil-transmitted helminths). The parasitic infection with the highest prevalence is urinary schistosomiasis, caused by flukes (*Schistosoma hematobium*), and the programs are planned to control this disease (*2*).

**Figure 1:**
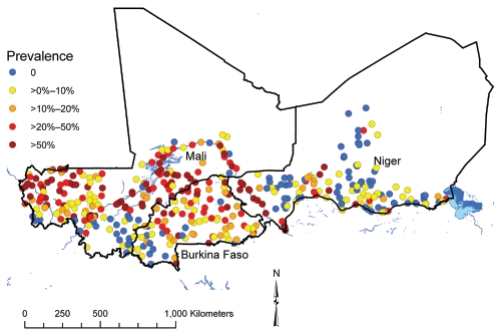
Prevalence of infection with *Schistosoma hematobium* at 418 survey locations in Burkina Faso, Mali, and Niger, 2004–2006.

Parasitologic data were collected in coordinated school-based field surveys in Burkina Faso, Mali, and Niger (Figure 1) during 2004–2006 (preintervention) by using standardized protocols (available on request). The collated dataset covered a spatially contiguous area, ≈2,750 km × 850 km, and included the infection status of 27,939 school-age children in 418 randomly selected locations. Infection status was defined according to egg count determined by microscopic examination of urine samples; >1 *S. hematobium* eggs indicated infection.

Spatial prediction was based on a logistic regression model (Table), constructed by using the software WinBUGS, version 1.4.2 (MRC Biostatistics Unit, Cambridge and Imperial College, London, UK). The model had infection status as the binary outcome variable, age and sex of the survey participants as individual-level fixed effects, and distance from perennial water body (derived from electronic maps obtained from the Food and Agriculture Organization) and land surface temperature (LST; with a quadratic term; see Hay et al. [*10*] for details on how these data were derived) as survey location–level fixed effects. Variable selection methods and the model are presented in the online Technical Appendix. The model also included a geostatistical random effect for residual spatial clustering of infection prevalence (*11*).

**Table:** Bayesian logistic regression model of prevalence of infection with *Schistosoma haematobium* in children in 418 schools in Burkina Faso, Mali, and Niger, 2004–2006*

Variable	Posterior distribution	
	Mean (95% CrI)	SD
Female gender	0.70 (0.65–0.76)	0.03
Age, y		
9–10	1.16 (1.00–1.33)	0.08
11–12	1.51 (1.31–1.73)	0.10
13–16	1.79 (1.53–2.06)	0.14
Distance to perennial water body	0.34 (0.21–0.54)	0.08
Land surface temperature	0.80 (0.51–1.21)	0.18
Land surface temperature^2^	1.10 (0.85–1.40)	0.14
Rate of decay of spatial correlation	2.03 (1.48–2.74)	0.32
Variance of the spatial random effect (sill)	7.03 (5.36–9.31)	1.01

*CrI, Bayesian credible interval. Values for the fixed effects are odds ratios; note the odds ratios for the climate variables are on a common scale, where the variables were standardized to have a mean = 0 and SD = 1. The reference group for sex was boys and for age was 6–8 y. The number of children found to be infected with *S. haematobium* was modeled by using a binomial distribution described by the proportion infected and the total number sampled in each survey location. The proportion infected was modeled by using logistic regression with an intercept, covariates (sex, age, distance to perennial water body, land surface temperature, and a quadratic term for land surface temperature), and a random effect that described spatial correlation (i.e., clustering). Model outputs were distributions (termed *posterior distributions*) that can be summarized by using the mean, SD, and 95% CrI (representing the range of values that contains the true value with a probability of 95%). More details on the model are presented in the Technical Appendix.

A prevalence map for the study area was constructed, using the model, by predicting infection prevalence at the centroids of cells of a 0.15 × 0.15 decimal degree (≈18 km × 18 km) grid. This model was implemented with the *spatial.unipred* command of WinBUGS (details are provided in the Technical Appendix). Estimates from Bayesian models are distributions (termed posterior distributions) that represent the probability of each of a range of plausible values being true for the variable being modeled. To quantify the uncertainties surrounding the model predictions, we plotted the probability of each prediction location having a prevalence >50%, rather than mean predicted prevalence at each location. The probabilities were calculated from the posterior distributions of predicted prevalence at each location (i.e., if 95% of the posterior distribution of predicted prevalence was >0.5, the probability of prevalence >50% at that location was 95%).

Cross-validation was done by randomly allocating survey locations to 3 groups and undertaking 3 separate runs of the model; 1 of the 3 groups was sequentially omitted, and predicted prevalence at the omitted locations was determined by using the model. Predicted prevalence was compared with observed prevalence, dichotomized, according to a 50% observed prevalence threshold. The comparison statistic was the area under the curve (AUC) of the receiver operating characteristic, and a value of >0.7 was considered to indicate acceptable predictive ability. An average AUC was calculated across the 3 model runs.

In the final model (Table), statistically significant correlations suggested that infection prevalence was higher in older boys and increased with proximity to perennial bodies of water, but no association was found between prevalence and LST. The range over which spatial correlation was >5% (chosen to indicate statistically important spatial correlation) was ≈177 km, indicating the approximate radius of clusters. Results of the validation analysis showed an average AUC of 0.86, indicating that the model had an acceptable predictive performance.

**Figure 2:**
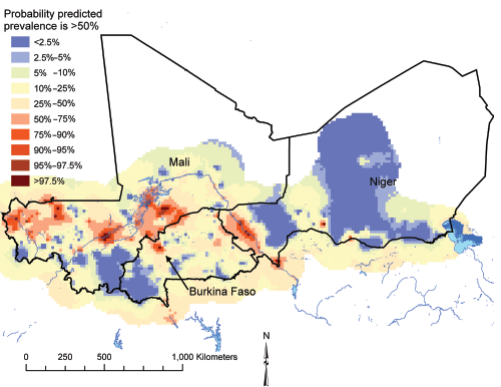
Predicted probability of prevalence of infection with *Schistosoma hematobium* being >50% in Burkina Faso, Mali, and Niger in boys ages 13–16 years; results are based on a Bayesian geostatistical model. The red areas had a low degree of uncertainty that predicted prevalence was >50%, and the blue areas had a high degree of uncertainty that predicted prevalence was >50%.

Bayesian probability maps were produced for each sex and age group, but for illustrative purposes we present predicted probability of prevalence >50% in boys ages 13–16 years (the group with the highest infection prevalence; Figure 2). Large clusters of prediction locations with a high probability (i.e., >50%; indicative of low uncertainty) of prevalence being >50% intervention threshold were located in a mid-latitudinal band across Mali, running from western to central regions, and in the Niger River valley region of Niger. Smaller clusters were located in various parts of southern and eastern Mali, northwestern and northeastern Burkina Faso, and south-central Niger.

## Conclusions

Future schistosomiasis control plans should acknowledge uncertainties such as those presented in Figure 2. A possible approach would be to introduce a second threshold for the level of uncertainty that a location is above the intervention prevalence threshold; if the uncertainty is greater than this second threshold, then the location is excluded until new evidence is obtained that confirms prevalence is above or below the intervention prevalence threshold. This second uncertainty threshold should be determined by the quantity of resources available for disease control and the level of decision risk deemed appropriate.

In addition to providing an evidence base for distributing resources in 3 West African countries as part of the SCI-supported national control programs, the maps presented here have a potential role in maintaining sustainability of schistosomiasis control after SCI support ends (SCI is funded through 2009). They can be used as advocacy tools for channeling funds to high-risk populations in the affected countries and, in the likely event that money for schistosomiasis control in these countries becomes more limited after SCI support ends, they can be used to ensure that scarce governmental resources are distributed as efficiently as possible. National coordinators who might face accountability for targeted (i.e., unequal) distribution of resources will benefit from the defendable, scientifically sound methods presented in this article. By focusing on uncertainty in spatial predictions, more flexible tools for disease control can be developed that allow the geographic dimensions of control programs to be scaled and modified according to available resources and acceptable levels of decision risk.

## Supplementary Material

Technical Appendix Mapping the Probability of Schistosomiasis and Associated Uncertainty, West Africa
