# Supplementary material for: Mapping the Probability of Schistosomiasis and Associated Uncertainty, West Africa
Source: Emerg Infect Dis. 2008 Oct;14(10):1629–32. doi: 10.3201/eid1410.080366 (PMC2609896; doi:10.3201/eid1410.080366)
Supplement: Technical Appendix — Mapping the Probability of Schistosomiasis and Associated Uncertainty, West Africa [file 08-0366_Techapp-s1.pdf]

# Mapping the Probability of Schistosomiasis and Associated Uncertainty, West Africa

## Technical Appendix

### Variable Selection

Covariates were initially selected on the basis of biological plausibility. Age and sex have both been demonstrated to be associated with urinary schistosomiasis prevalence likely due to physiologic differences in susceptibility. Transmission of *Schistosoma haematobium* involves contact with cercariae in water in which the intermediate host snail, *Bulinus* spp., is present. Proximity to a perennial water body is, therefore, a plausible risk factor for exposure and subsequent infection. The intermediate host snail has an optimal temperature range above and below which survival is impeded, justifying the inclusion of a quadratic term for land surface temperature (LST). Precipitation levels might indicate the availability of snail habitats and (in high-precipitation areas) the risk of washout of snail populations; normalized difference vegetation index (NDVI) is a proxy measure of rainfall that was also considered for inclusion in the model. Elevation was not considered for inclusion because of collinearity with temperature and precipitation. Therefore, the initial candidate set of variables included sex, age, proximity to perennial water bodies, LST (with and without a quadratic term) and NDVI. Variable selection was done in Stata/SE 10.0 (StataCorp, College Station, TX, USA) by using a fixed-effects logistic regression model with backwards variable selection, with an exclusion criterion of Wald $p>0.2$. NDVI was excluded and all remaining variables were selected for inclusion in the spatial model. The quadratic term for LST was significant.

### Model Building and Assessment

The model was of the form

$$Y_{i,j} \sim Binomial(n_{i,j}, p_{i,j})$$

where $Y_{i,j}$ was the number of positive infection status individuals, $n_{i,j}$ was the number tested and $p_{i,j}$ was the risk for positive infection status in age-gender group $i$, location $j$;

$$logit(p_{i,j}) = \alpha + \beta \times girl_{i,j} + \delta 1 \times age1_{i,j} + \delta 2 \times age2_{i,j} + \delta 3 \times age3_{i,j} + \lambda_j$$

where $\alpha$ was the intercept, $\beta$ was the coefficient for female gender, $\delta$1-3 were the coefficients for age groups 9–10, 11–12, and 13–16 years;

$$\lambda_j = \varepsilon \times dist_j + \gamma \times LST_j + \kappa \times LST_j^2 + \theta_j$$

where $\varepsilon$ was the coefficient for distance to perennial water body, $\gamma$ was the coefficient for land surface temperature (LST), $\kappa$ was the coefficient for the quadratic term of LST; and $\theta_j$ was defined by the isotropic, exponentially decaying correlation function

$$f(d_{ij};\phi) = \exp\left[-(\phi d_{ij})\right]$$

where $d_{ij}$ are the distances between pairs of points $i$ and $j$, and $\phi$ is the rate of decline of spatial correlation per unit of distance. Noninformative priors were specified for the intercept (uniform prior with bounds $-\infty$ and $\infty$) and the coefficients (normal prior with mean = 0 and precision, the inverse of variance, = $1 \times 10^{-4}$). The prior distribution of $\phi$ was also uniform with upper and lower bounds set at 0.1 and 50. The precision of $\theta_j$ was given a noninformative prior gamma distribution.

#### Implementation of the *spatial.unipred* Command in WinBUGS

In WinBUGS, interpolation of a variable to nonsampled locations can be done using the *spatial.unipred* command, where the spatial correlation structure of that variable has been modeled by using an exponentially decaying correlation function fitted to the observed data. The *spatial.unipred* interpolation function uses kriging, where predicted values are a weighted average of observed data obtained from nearby locations with weights being apportioned according to distance and direction from the prediction location. In our models, the interpolated variable was the spatial random effect. Predicted prevalences were calculated by adding the interpolated random effect to the sum of the products of the coefficients for the fixed effects and the values of the fixed effects at each prediction location. For the individual-level fixed effects (sex and age), separate calculations were done, where the coefficient for the relevant age and sex were added to the sum. The overall sum was then back-transformed from the logit scale to the prevalence scale, giving prediction surfaces for prevalence of infection in each age and sex group.
